# Author Correction: Deep learning radiomics can predict axillary lymph node status in early-stage breast cancer

**DOI:** 10.1038/s41467-021-24605-8

**Published:** 2021-07-12

**Authors:** Xueyi Zheng, Zhao Yao, Yini Huang, Yanyan Yu, Yun Wang, Yubo Liu, Rushuang Mao, Fei Li, Yang Xiao, Yuanyuan Wang, Yixin Hu, Jinhua Yu, Jianhua Zhou

**Affiliations:** 1 Department of Ultrasound, Sun Yat-Sen University Cancer Center, State Key Laboratory of Oncology in South China, Collaborative Innovation Center for Cancer Medicine, Guangzhou, China; 2 Department of Electronic Engineering, Fudan University, Shanghai, China; 3 Paul C. Lauterbur Research Center for Biomedical Imaging, Institute of Biomedical and Health Engineering, Shenzhen Institutes of Advanced Technology, Chinese Academy of Sciences, Shenzhen, China; 4 The key laboratory of medical imaging computing and computer assisted intervention of Shanghai, Shanghai, China

**Keywords:** Breast cancer, Cancer imaging, Cancer screening, Oncology

Correction to: *Nature Communications* 10.1038/s41467-020-15027-z, Published online 06 March 2020.

This Article contained an error in the Supplementary Software. The deep learning radiomics software for predicting axillary lymph node status was damaged when the files were compressed during the submission process. The original code availability statement stated that the code was provided in two separate files, the code is now provided in one file. The software has now been corrected and the Code Availability statement has been corrected to read ‘The software and code of the proposed method are available as Supplementary Software’.

## Supplementary information

Supplementary software

