# Supplementary material for: Deep learning radiomics can predict axillary lymph node status in early-stage breast cancer
Source: Nat Commun. 2020 Mar 6;11:1236. doi: 10.1038/s41467-020-15027-z (PMC7060275; doi:10.1038/s41467-020-15027-z)
Supplement: Supplementary file 1 — Supplementary Information [file 41467_2020_15027_MOESM1_ESM.pdf]

Deep learning radiomics can predict axillary lymph node status in early-stage breast cancer

Zheng et al.

Supplementary methods

Network model parameters. The DLR model is mainly based on ResNet50 and inherits all convolution layers of it. Globally, one path of the DLR model is stacked from a 224*224*3 input layer, a 7*7 convolutional layer, a max-pooling layer and a series of residual blocks, following a global average pooling (GAP) layer and three fully connected (FC) layers with 128, 64 and 2 neurons, respectively. For the 2D-SWE and US ROIs inputs, a parallel path is added and both share parameters. The two modal images are encoded by GAP layer and then concatenated as the input of the following FC layers. The detailed structure of the network is shown in Supplementary Table 1.

Supplementary Table 1. The detailed parameters of the network

Layer name	Output size	DLR
Conv1	112×112	$7\times7, 64, \text{stride } 2$
Conv2_x	56×56	$\begin{bmatrix}1\times1, 64\\3\times3, 64\\1\times1, 256\end{bmatrix}\times3$
Conv3_x	28×28	$\begin{bmatrix}1\times1, 128\\3\times3, 128\\1\times1, 512\end{bmatrix}\times4$
Conv4_x	14×14	$\begin{bmatrix}1\times1, 256\\3\times3, 256\\1\times1, 1024\end{bmatrix}\times6$
Conv5_x	7×7	$\begin{bmatrix}1\times1, 512\\3\times3, 512\\1\times1, 2048\end{bmatrix}\times3$
Fc6	1×1	$\text{Average pool}, 256-\text{d fc}$
Fc7	1×1	$64-\text{d fc}$
Fc8	1×1	$2-\text{d fc}, \text{softmax}$

*Note: the gray shading (Fc7) refers to deep feature output layer.

Statistic metric. The following 6 measurements including area under the receiver operating characteristic curve (AUC), accuracy, sensitivity, specificity, positive predictive value (PPV) and negative predictive value (NPV) were

calculated to evaluate the model performance.

1、 **AUC:**

$$\mathrm{AUC} = \frac{\sum_{ins_i \in positiveclass} rank_{ins_i} - \frac{M \times (M+1)}{2}}{M \times N}$$

Where M, N are the number of positive samples and negative samples respectively. $rank_{ins_i}$ is the serial number of sample i. $\sum_{ins_i \in positiveclass}$ means add up the serial numbers of the positive samples.

2、 **accuracy:**

$$\mathrm{accuracy} = \frac{TP + TN}{TP + TN + FP + FN}$$

3、 **sensitivity:**

$$\mathrm{sensitivity} = \frac{TP}{TP + FN}$$

4、 **specificity:**

$$\mathrm{specificity} = \frac{TN}{TN + FP}$$

5、 **positive predictive value:**

$$\mathrm{PPV} = \frac{TP}{TP + FP}$$

6、 **negative predictive value:**

$$\mathrm{NPV} = \frac{TN}{TN + FN}$$

Where TP is true positive, TN is true negative, FP is false positive and FN is false negative.

Supplementary Note 1

The experiment results of just using the US or the SWE combined with clinical parameters to predict ALN metastasis (N0 vs N_+(≥1)) are shown in Supplementary Table 2.

Supplementary Table 2. The results of using single modal image and clinical parameters to predict ALN metastasis (N0 v.s. N_+(≥1))

Methods		AUC	ACC (%)	SENS (%)	SPEC (%)	PPV (%)	NPV (%)
Experiment1	T	0.800 [0.757, 0.843]	72.2 [67.3, 76.7]	71.6 [64.7, 77.8]	73.8 [67.1, 79.7]	72.7 [65.8, 78.8]	72.7 [66.0, 78.7]
	V	0.767 [0.675, 0.858]	71.6 [63.3, 79.8]	67.4 [52.5, 80.1]	73.1 [60.9, 83.2]	64.7 [50.1, 77.6]	75.4 [63.1, 85.2]
	I-T	0.752* [0.657, 0.847]	72.0 [63.0, 79.4]	63.3 [48.3, 76.6]	79.4 [67.9, 88.3]	68.9 [53.4, 81.8]	75.0 [63.3, 84.5]
Experiment2	T	0.790 [0.745, 0.834]	72.2 [67.3, 76.7]	73.1 [66.3, 79.2]	71.8 [65.0, 77.9]	71.6 [64.9, 77.8]	73.2 [66.5, 79.3]
	V	0.750 [0.653, 0.846]	69.8 [60.5, 77.4]	71.4 [56.7, 83.4]	68.7 [56.2, 79.4]	62.5 [48.5, 75.1]	76.7 [63.8, 86.7]
	I-T	0.731** [0.636, 0.826]	68.6 [59.4, 76.2]	70.0 [55.4, 82.1]	63.2 [50.7, 74.6]	58.3 [44.9, 70.9]	74.1 [61.0, 84.7]

Note: Experiment1 = US + clinical parameters; Experiment2 = SWE + clinical parameters. 95% confidence intervals are included in brackets. AUC=area under the receiver operating characteristic curve; ACC=accuracy; SENS=sensitivity; SPEC=specificity; PPV= positive predict value; NPV= negative predict value; PARAM= model parameters amount; T = training cohort (n=350); V = validation cohort (n=116), I-T = independent test cohort (n=118).

* indicates *P*=0.006, Delong et al. in comparison with clinical parameter combined DLR in independent test cohort.

** indicates *P*=0.002, Delong et al. in comparison with clinical parameter combined DLR in independent test cohort.
