# Supplementary material for: Deep learning radiomics can predict axillary lymph node status in early-stage breast cancer
Source: Nat Commun. 2020 Mar 6;11:1236. doi: 10.1038/s41467-020-15027-z (PMC7060275; doi:10.1038/s41467-020-15027-z)
Supplement: Supplementary file 3 — Description of Additional Supplementary Files [file 41467_2020_15027_MOESM3_ESM.pdf]

Description of Additional Supplementary Files

File Name: Supplementary Software 1
Description: ZIP file containing software used to predict axillary lymph node status of breast cancers based on deep learning radiomics of the conventional ultrasound images and shear wave elastography images of the breast lesions. Software 1 is the main file of the software and Software 2 is the executive program (.exe file.) of the software.
