# Supplementary material for: Deep learning radiomics can predict axillary lymph node status in early-stage breast cancer
Source: Nat Commun. 2020 Mar 6;11:1236. doi: 10.1038/s41467-020-15027-z (PMC7060275; doi:10.1038/s41467-020-15027-z)
Supplement: Supplementary file 5 — Reporting Summary [file 41467_2020_15027_MOESM5_ESM.pdf]

natureresearch

Corresponding author(s): Dr. Jianhua Zhou

Last updated by author(s): Jan 22, 2020

Reporting Summary

Nature Research wishes to improve the reproducibility of the work that we publish. This form provides structure for consistency and transparency in reporting. For further information on Nature Research policies, see Authors & Referees and the Editorial Policy Checklist .

Statistics

For all statistical analyses, confirm that the following items are present in the figure legend, table legend, main text, or Methods section.

n/a	Confirmed	
☐	☒	The exact sample size (*n*) for each experimental group/condition, given as a discrete number and unit of measurement
☐	☒	A statement on whether measurements were taken from distinct samples or whether the same sample was measured repeatedly
☐	☒	The statistical test(s) used AND whether they are one- or two-sided<br>*Only common tests should be described solely by name; describe more complex techniques in the Methods section.*
☒	☐	A description of all covariates tested
☒	☐	A description of any assumptions or corrections, such as tests of normality and adjustment for multiple comparisons
☐	☒	A full description of the statistical parameters including central tendency (e.g. means) or other basic estimates (e.g. regression coefficient) AND variation (e.g. standard deviation) or associated estimates of uncertainty (e.g. confidence intervals)
☐	☒	For null hypothesis testing, the test statistic (e.g. *F*, *t*, *r*) with confidence intervals, effect sizes, degrees of freedom and *P* value noted<br>*Give P values as exact values whenever suitable.*
☒	☐	For Bayesian analysis, information on the choice of priors and Markov chain Monte Carlo settings
☐	☒	For hierarchical and complex designs, identification of the appropriate level for tests and full reporting of outcomes
☒	☐	Estimates of effect sizes (e.g. Cohen's *d*, Pearson's *r*), indicating how they were calculated

Our web collection on statistics for biologists contains articles on many of the points above.

Software and code

Policy information about availability of computer code

Data collection	Siemens S2000 ultrasound scanner equipped with a 4–9MHz linear array transducer.
Data analysis	MedCalc software (V.11.2; 2011 MedCalc Software bvba, Mariakerke, Belgium); Matlab R2015b; Python 3.5.

For manuscripts utilizing custom algorithms or software that are central to the research but not yet described in published literature, software must be made available to editors/reviewers. We strongly encourage code deposition in a community repository (e.g. GitHub). See the Nature Research guidelines for submitting code & software for further information.

Data

Policy information about availability of data

All manuscripts must include a data availability statement. This statement should provide the following information, where applicable:

- Accession codes, unique identifiers, or web links for publicly available datasets
- A list of figures that have associated raw data
- A description of any restrictions on data availability

Excel files containing raw data included in the main figures and tables can be found in the Source Data File. The imaging data can be provided upon request to the corresponding author.The software and code of the proposed method have been separated into two files and uploaded as Supplementary Information file in the Article. The software of the proposed method can also be obtained at https://drive.google.com/open?id=1qdzO5O6jP_l16AiZB-y0zWVEx2eCtJqq.

Field-specific reporting

Please select the one below that is the best fit for your research. If you are not sure, read the appropriate sections before making your selection.

Life sciences study design

All studies must disclose on these points even when the disclosure is negative.

Sample size	The inclusion criteria included the followings: (a) women with US-suspected breast masses; (b) availability of clinical data; (c) patients who underwent breast surgery and sentinel lymph node biopsy or ALN dissection with curative intent. Finally 584 women with 584 malignant breast lesions were enrolled for analysis.
Data exclusions	(a) patients who received preoperative therapy (resection biopsy, neoadjuvant radiotherapy or chemotherapy) because preoperative therapy might influence the breast mass and the status of ALN; (b) patients with multifocal lesions or bilateral disease because it is hard to determine which lesion should be chosen to input into the software; (c) mass deeper than 3 cm in depth due to the attenuation of SWE or larger than 3.5 cm in diameter due to the limited width of the US probe; (d) unqualified 2D-SWE measurements, which means little or no shear wave signal was acquired in the ROI of SWE; These images are unqualified. (e) benign breast lesions or carcinoma in situ because these cases would not have ALN metastasis; (f) cases missing important pathological results (immunohistochemical results or lymph node results) because all this pathological results are needed to input into the software; (g)cases with incomplete information or images because some information and images are needed to input into the software.
Replication	Models were verified and replicated using regular machine learning metrics on independent test cohorts. We released the software of the model for replication on new data.
Randomization	The enrolled samples were randomly divided into the training cohort and test cohort.
Blinding	The investigator were blinded to group allocation during data collection and analysis.

Reporting for specific materials, systems and methods

We require information from authors about some types of materials, experimental systems and methods used in many studies. Here, indicate whether each material, system or method listed is relevant to your study. If you are not sure if a list item applies to your research, read the appropriate section before selecting a response.

Materials & experimental systems

n/a	Involved in the study	
☒	☐	Antibodies
☒	☐	Eukaryotic cell lines
☒	☐	Palaeontology
☒	☐	Animals and other organisms
☐	☒	Human research participants
☐	☒	Clinical data

Methods

n/a	Involved in the study	
☒	☐	ChIP-seq
☒	☐	Flow cytometry
☒	☐	MRI-based neuroimaging

Human research participants

Policy information about studies involving human research participants

Population characteristics	584 women (mean age, 50 years; range, 26–83 years) with 584 malignant breast lesions were analysed, among them 337 had disease-free axilla, 150 had low metastatic burden of axillary disease and 97 had heavy metastatic burden of axillary disease.
Recruitment	Patients with breast lesions were recruited in Sun Yat-sen University Cancer Center. There was no potential self-selection bias to impact results.
Ethics oversight	This prospective study was approved by Institutional Review Board of Sun Yat-sen University Cancer Center.

Note that full information on the approval of the study protocol must also be provided in the manuscript.

Clinical data

Policy information about clinical studies

All manuscripts should comply with the ICMJEguidelines for publication of clinical research and a completedCONSORT checklist must be included with all submissions.

Clinical trial registration	ChiCTR1900028461 from Chinese Clinical Trial Registry
Study protocol	The study protocol can be assessed at Chinese Clinical Trial Registry.
Data collection	Between January 2016 and April 2019, a total of 1342 women with 1342 breast lesions was studied in Sun Yat-sen University Cancer Center and finally 584 women (mean age, 50 years; range, 26–83 years) with 584 malignant breast lesions were enrolled for analysis. Radiologists performed preoperative breast conventional US, breast SWE, and axillary conventional US. Clinical and

histopathologic data were obtained from the medical records.

Outcomes

We assess different methods by calculating the diagnostic performances in predicting ALN status between disease-free axilla (N0) and any axillary metastasis (N+(≥1)), between low metastatic burden of axillary disease (N+(1-2)) and heavy metastatic burden of axillary disease (N+(≥3)).
